# Syndrome of arachnomelia in Simmental cattle

**DOI:** 10.1186/1746-6148-4-39

**Published:** 2008-10-01

**Authors:** Johannes Buitkamp, Bernhard Luntz, Reiner Emmerling, Horst-Dieter Reichenbach, Myriam Weppert, Benjamin Schade, Norbert Meier, Kay-Uwe Götz

**Affiliations:** 1 Bavarian State Research Center for Agriculture, Institute of Animal Breeding, 85580 Grub, Germany; 2 Institute of Molecular Animal Breeding and Biotechnology, Gene Center of the Ludwig-Maximilian University, 81377 Munich, Germany; 3 Bavaria Animal Health Service, Department of Pathology, 85586 Grub, Germany

## Abstract

**Background:**

The syndrome of arachnomelia is an inherited malformation mainly of limbs, back and head in cattle. At present the arachnomelia syndrome has been well known mainly in Brown Swiss cattle. Nevertheless, the arachnomelia syndrome had been observed in the Hessian Simmental population during the decade 1964–1974. Recently, stillborn Simmental calves were observed having a morphology similar to the arachnomelia syndrome. The goal of this work was the characterization of the morphology and genealogy of the syndrome in Simmental to establish the basis for an effective management of the disease.

**Results:**

The first pathologically confirmed arachnomelia syndrome-cases in the current Simmental population appeared in the year 2005. By 2007, an additional 140 calves with the arachnomelia syndrome were identified. The major pathological findings were malformed bones affecting the head, long bones of the legs and the vertebral column. It could be shown that, with the exception of two cases that were considered as phenocopies, all of the paternal and about two-third of the maternal pedigrees of the affected calves could be traced back to one common founder. Together with the data from experimental matings, the pedigree data support an autosomal recessive mutation being the etiology of the arachnomelia syndrome. The frequency of the mutation in the current population was estimated to be 3.32%.

**Conclusion:**

We describe the repeated occurrence of the arachnomelia syndrome in Simmental calves. It resembles completely the same defect occurring in the Brown Swiss breed. The mutation became relatively widespread amongst the current population. Therefore, a control system has to be established and it is highly desirable to map the disease and develop a genetic test system.

## Background

In the year 2006 a syndrome was described in the German and Austrian Simmental (Fleckvieh, as it is locally called, is the main dual-purpose breed in Germany, in short called Simmental in the further text) population, that was pathologically similar to the arachnomelia syndrome in Brown Swiss cattle [1]. The congenital arachnomelia syndrome (AS, OMIA Phene ID 139, Group 000059) is mainly a malformation of the skeletal system in cattle that was initially described by Rieck and Schade [2] in Holstein Friesian, Red Holstein and Simmental.

The main pathological changes are skeletal malformations of the legs, the spinal column and the skull. The legs are thinner and appear longer than normal (dolichostenomelia, arachnomelia) since the diameter of the diaphyses is reduced. These long bones are more fragile and, in combination with stiffened joints, they tend to fracture during calving. The fetlock joints are deformed, often stiffened and show hyperextension. The malformation of the spinal column leads to kyphosis and scoliosis. The skull malformations are characterized by a shortened lower jaw (brachygnathia inferior), convex rounding of the frontal bone leading to a marked stop ("pointer head") and rotation of the anterior cranium. In some cases, additional malformations like hydrocephalus externus develop [2-5].

Since the report of Rieck and Schade [2] no further cases were reported in Simmental cattle, but in the 1980s the syndrome was dispersed in another breed, the European Brown Swiss cattle, by the use of American Brown Swiss sires [4,6]. In Brown Swiss an autosomal recessive mode of inheritance was supposed and a control program based on the identification of carriers by pedigree analyses was established [5]. Recently, four cases of arachnomelia syndrome were reported in Italy [3].

In this study, we present the data of 152 pathologically confirmed cases of arachnomelia syndrome in Simmental that were collected from October 2005 to March 2007. We describe the pathological findings, the familial occurrence and an estimate of the frequency of the diseases allele in Simmental cattle. Additional support for the mode of inheritance and the genetic basis of the arachnomelia syndrome is given by the result of experimental matings of obligate carriers.

## Results and discussion

AS has not been reported again in Simmental since its first description in the 1970s, more than thirteen years ago. In autumn 2004 a number of stillborn calves with similar malformations of the legs and head were recorded within the monitoring system of anomalies in Simmental. Some of these calves were sent to the veterinary service laboratory for examination, and in December 2005 the first 15 cases of AS were pathologically confirmed. Subsequently, farmers and veterinarians had been encouraged to report cases by an information leaflet and various articles in local trade journals. An increasing number of suspected cases was reported and an additional 136 affected calves were identified by pathological examination by June 2007.

### Familial occurrence and case presentation of the syndrome of arachnomelia

**Figure 1:**
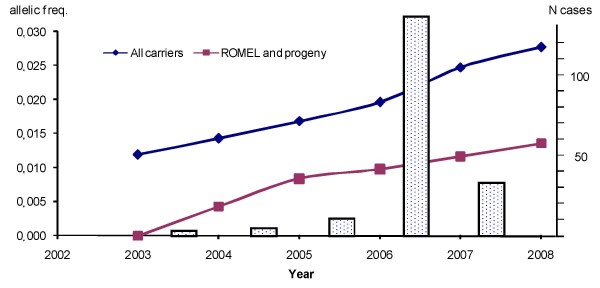
**Allelic frequency and number of confirmed cases of AS**. The allelic frequency of the AS carriers in the living cows of the breeding population is shown per year (blue line). The lower line (red) shows the frequency estimation based on ROMEL and his progeny (including females, ignoring other carriers). The number of pathologically confirmed cases per year is shown by bars.

**Figure 2:**
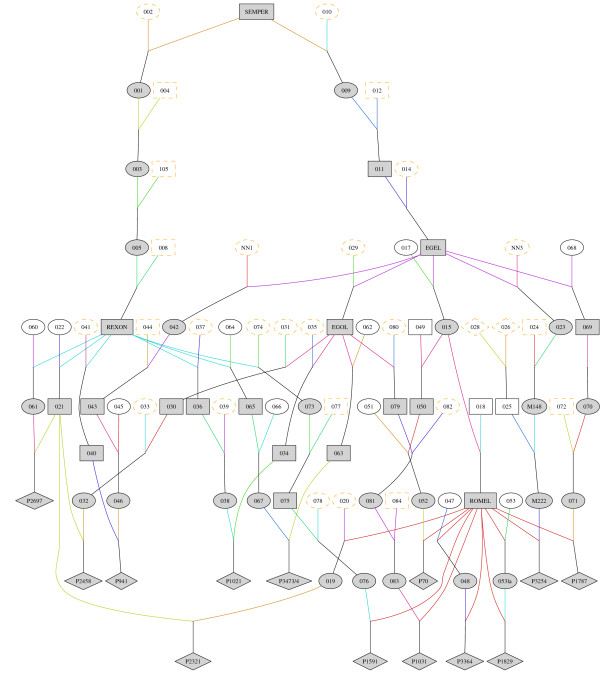
**Extracted pedigree of AS affected calves**. Part of the pedigree that shows the inheritance path of the AS mutation from SEMPER to the first 13 affected calves. Individuals that were most likely carriers of the AS mutation are shown in gray, males as square, females as oval, affected calves as rhomb.

The geographical origins of the cases were the southern part of Germany and Austria, reflecting the regional distribution of the Simmental breed. Both sexes were equally represented in the 152 (80 male, 72 female, χ^2 ^= 0.21, p = 0.64) affected calves. The largest number of cases was registered in 2006 (Figure 1). In retrospect, it could be shown that the main reason for the rapid increase of cases in the years 2005 and 2006 was the high popularity of certain sires carrying the AS mutation (ROMEL, ISO-Nr. 276000911043667, born in 1995; EGEL, 276000915512806, 1985; REXON, 276000913008210, 1989). The latter two sires represent the key-nodes of the pedigree pathways of the mutation from the founder into the current population (Figure 2). ROMEL, for example, sired more than 40,000 cows 4 to 6 years ago. Furthermore, 115 sons of ROMEL born from 2001 to 2005 are registered and listed in the breeding database [7]. These progeny were now mated to ROMEL and sons or grandsons of EGEL and REXON resulting in a high probability for the occurrence of affected calves. Increasing awareness of the disease and abandoning of selling the semen from carriers led to a sharp drop of cases in 2007. The disease was successfully managed by efficient collaboration of the Institute for Animal Breeding of the Bavarian State Research Centre for Agriculture (LfL), the Landeskuratorium der Erzeugerringe für tierische Veredelung in Bayern e.V (LKV), the Bavarian Animal Health Service (TGD) and breeding organizations.

### Pathological findings

**Figure 3:**
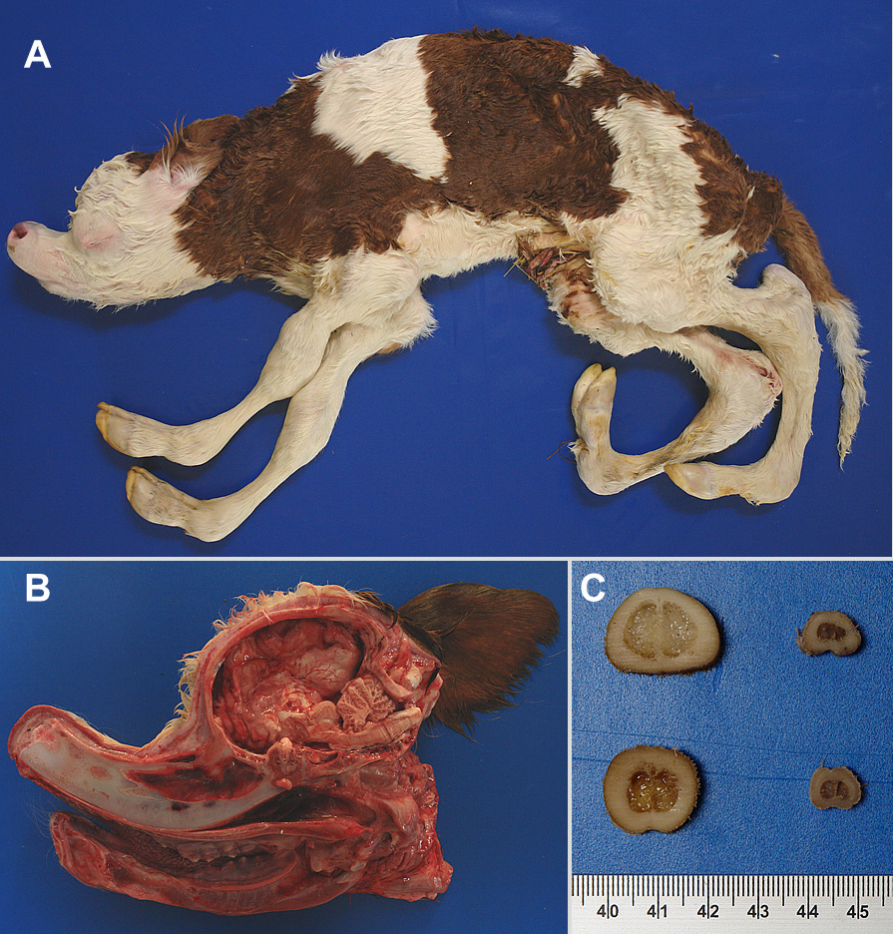
**An AS affected calf with typical pathological findings**. A – Overview: Note dolichostenomelia, convexity of the frontal bone, and kyphosis. B – Sagittal section through the head: Severe deformation (dent forming, brachygnathia inferior, compression of cerebrellum). C – Cross sections of metacarpus (on the top) and -tarsus (at the bottom); sections of an unaffected calf as a control on the left. Note the dysplasia of the bone of the diaphysis. It is mainly due to a decreased diameter whereas the width of the substantia compacta is normal.

Calves under suspicion of the arachnomelia syndrome were sent to the pathology department of the TGD for macroscopic examination. The observed major pathological findings were (1) facial deformation, including brachygnathia inferior and concave rounding of the maxilla forming a dent ('pointer-head'); (2) abnormally thin diaphyses of the long bones (the outer diameter of the diaphyses is diminished, whereas the width of the substantia compacta is normal) leading to frequent fractures of the metacarpus and metatarsus in the course of forced birth assistance ('spider-legs', dolichostenomelia). The deformations of other bones of the legs were less apparent and the scapula was usually unaffected; (3) angular deformations of the distal parts of the legs characterized by bilateral stiff and hyperextended fetlocks with the extremity of the toe forward and parallel to the trunk of the body; and (4) defects of the vertebral column (kyphosis and scoliosis), but not of the ribs (Figure 3A–C). Additionally, inconsistent pathological findings included cerebral herniation combined with a malformed foramen magnum, microphthalmia, and external and internal hydrocephalus. The latter seem to develop secondarily, due to the enlarged foramen magnum.

Histological examination of selected cases revealed the presence of hemorrhages at the osteochondral junction of the epiphysis and an abrupt transmission from chondral to osteogenic tissue.

Cases never showed isolated malformations, e.g. of the head or legs, but usually a combination of all pathological findings that are characteristic for the syndrome. Nevertheless, the degree of the lesions ranged from obvious spider-leg cases to moderate or mild changes, making a definite diagnosis difficult. The latter cases (3) were excluded from the initial pedigree analyses. Meanwhile, an indirect gene test is available that has been developed at the Institute for Animal Breeding of the Bavarian State Research Centre for Agriculture (ITZ) and it could be shown that these cases are most probably not genetically affected (Buitkamp *et al*., in preparation).

### Carrier identification

Two criteria were used for carrier identification. The first was the presence of a calf that was diagnosed by pathological investigation. In many cases more than one affected calf per sire was identified [8]. Some sires had only one affected calf, but a large number of risk-matings. In these cases a second criterion, the statistical evaluation of risk-matings, was used to identify potential phenocopies. For this purpose, the probability of observing only a single affected calf among a certain number of risk-matings of the sire in question was calculated. Risk-matings were defined as matings with direct progenies of identified AS carriers. The probability of observing an affected calf depends also on the probability that such a calf is reported to the LKV. We assumed this probability to be 50%. Under these conditions, the probability of observing only one affected calf is lower than 1.0 percent, if at least 104 risk-matings are given for a single sire. In this case it is very likely that the single affected calf is a phenocopy. In 2006 and 2007 this was the case for two sires used for artificial insemination that had no pedigree connection to SEMPER (see below).

### Experimental matings of obligate carriers

**Figure 4:**
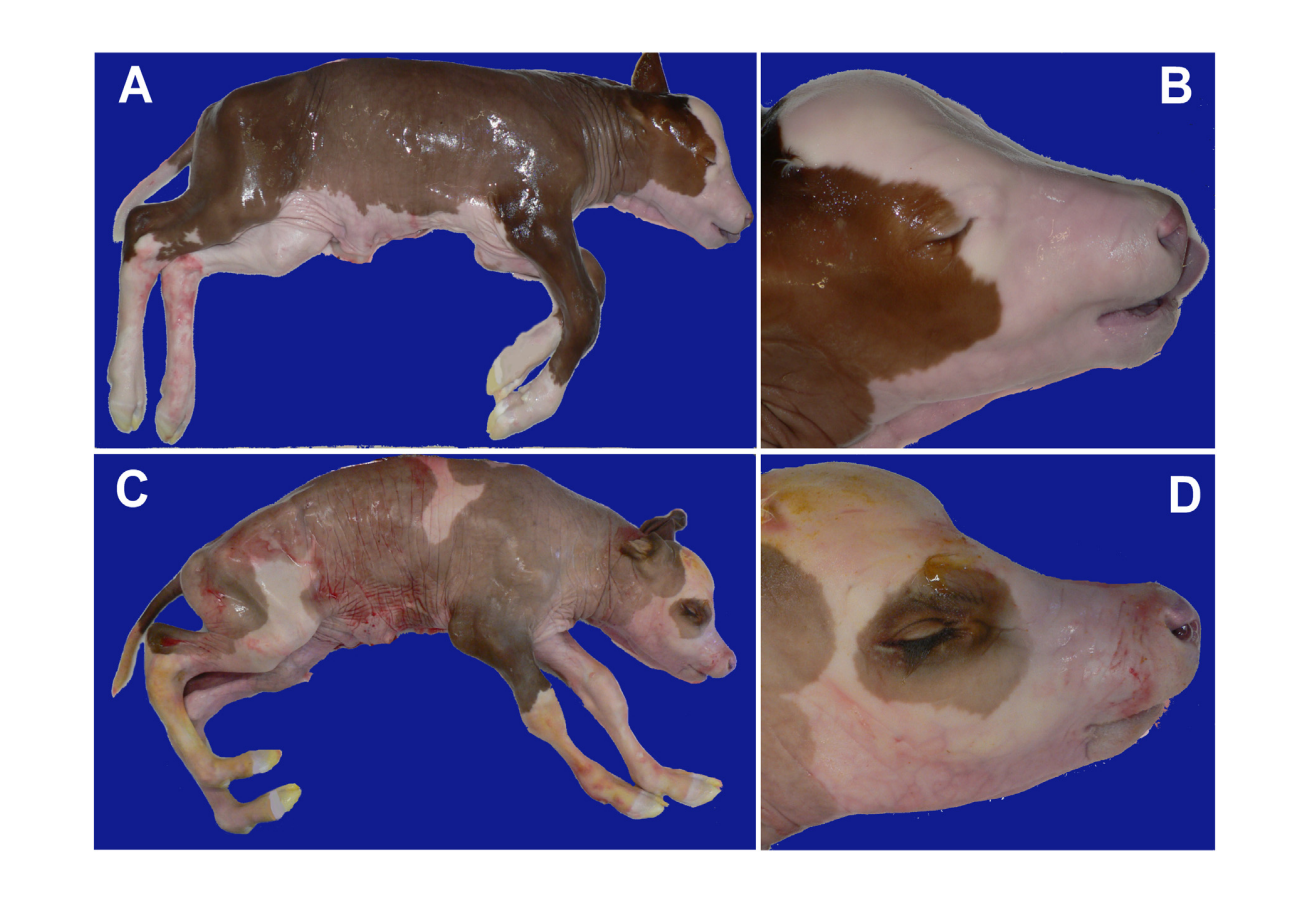
**225-days old fetuses from experimental matings of obligate arachnomelia syndrome carriers**. A, B – Normal 225-day fetus, overview and head. C, D – AS affected 225-day fetus, overview and head.

Four out of seven cows that were known AS carriers brought to the facilities of the ITZ were used for embryo transfer (Table 1). 33 of the 60 recipients (55%) were confirmed pregnant on day 35. Four of the 33 pregnant heifers (12%) aborted between days 36 and 49 of pregnancy. Of the remaining 29 recipients, 6 were slaughtered on day 150, 6 on day 200, and 17 animals on day 225 of pregnancy (Table 1). Four fetuses (three male and one female) out of 29 (14%) showed the typical pathological changes of the arachnomelia syndrome as described above (Figure 4C,D). All other fetuses showed no signs of AS (Figure 4A,B).

**Table 1:** Embryo production and transfer results using obligate carriers

							Fetuses				
							
		Transfers	Pregnancies (Day 35)		Abortions (Days 36–49)		Day* of pregnancy	AS unaffected		AS affected	
Donor	Sire	n	n	%	n	%		female	male	female	male
995	NAAB	9	7	(78)	1	(14)	225	2	2	1	1

997	NAAB	13	7	(54)	2	(29)	150	0	5	0	0
		10	6	(60)	0	-	200	1	5	0	0

998	ROMEL	6	1	(17)	0	-	150	0	1	0	0
		1	1	-	0	-	225	0	1	0	0
		8	3	(38)	0	-	225	1	2	0	0

082	LAND-MANN	8	5	(63)	1	(20)	225	1	3	0	0
		5	3	(60)	0	-	225	1	0	0	2

**Total**		**60**	**33**	**55**	**4**	**12**		**6**	**19**	**1**	**3**

*Day of slaughter or age of fetuses, respectively

Male fetuses represented 76% (22 of 29) of pregnancies (χ^2 ^= 3.123, p = 0.077, Yates corrected for sample size <30). Female weight (Table 2), crown-rump length at day 225 and chest circumference at day 225 of normal fetuses were lower than that of male fetuses. Fetuses that were affected by the arachnomelia syndrome showed lower weight than normal fetuses. The affected and the normal fetuses had similar crown-rump length, but the chest circumference of affected fetuses was higher than that of normal fetuses (Table 2). Due to the small number of affected female fetuses, a comparison with unaffected animals for sex was not possible. To compare unaffected and affected animals in total, the data were analyzed for statistical differences by the non parametric Mann-Whitney-Test. The only trait that was significantly different between unaffected and affected fetuses was the chest circumference (p = 0.004, Table 2). The body weight of AS affected calves was tendentially lower than that of normal calves. Since affected calves did not have different crown-rump-length and their chest circumference was even higher, this can best be explained by a reduced bone mass.

**Table 2:** Morphological data of 225 days old fetuses from experimental matings

	unaffected			affected			
	male	female	total	male	female	total	p*
N	8	5	13	3	1	4	
Average Fetus weight (kg)	20.74	19.02	20.08	19.15	17.90	18.84	0.141
Standard deviation	0.84	1.71	1.47	0.51	-	0.75	
Av. Crown – rump length (cm)	74.75	73.80	74.39	75.67	74.00	75.25	0.570
Standard deviation	1.98	1.30	1.76	3.06	-	2.63	
Av. Chest circumference (cm)	51.38	49.80	50.77	56.00	55.00	55.75	0.004
Standard deviation	1.60	1.64	1.74	1.00	-	2.86	

*p-value from the non parametric Mann-Whitney-Test for differences between unaffected and affected fetuses

### Pedigree Analysis and mode of inheritance

Eight-generation pedigrees of all cases were extracted from the joint German and Austria pedigree data and screened for common ancestors. The pedigree of the majority of affected calves (paternal line 150, maternal line 106 out of 152, Table 3) could be traced back to one founder, SEMPER (ISO-Nr. 27000979299305), a sire born in 1964, 6–9 generations before the affected calves were born (Figure 2). Most of the affected calves inherited the AS mutation via REXON or EGEL (Table 3, Figure 2). In 44 cases the maternal paths were not linked to the common pedigree (Table 3). One explanation would be the existence of additional, hereto unknown origins of the mutation. This could happen if the AS mutation is much more ancient and additional pedigree paths exist or if an independent mutation event happened leading to the same phenotype. An alternative, more plausible explanation could be the occurrence of misparentages. It is well known that in the pre parentage-test era, the frequency of false paternity, especially of the cows, was reasonable high (up to 23% [9]). Therefore, there is a good chance of a false registry within 6–9 generations.

**Table 3:** Pedigree connection of the arachnomelia syndrome-cases

**pedigree link**	**paternal**	**maternal**
SEMPER via EGEL	135	55
SEMPER via REXON	15	50
SEMPER via EGEL/REXON*	-	1
SENAT	-	2
no link to common carrier	2^†^	42
incomplete pedigree data	-	2

**Total**	**152**	**152**

The pedigree connection to the founder SEMPER (compare Figure 2) is given separately for the sire (paternal), and the cow (maternal) of the affected calve. They were listed separately for the two main lines, REXON and EGEL, that are progeny from SEMPER (3 and 4 generations away, respectively) and passed the mutation of the arachnomelia syndrome into the current population. Two maternal paths were not connected to SEMPER but to SENAT, the sire of SEMPER.

* Both sires, EGEL and REXON, appear in the pedigree

^†^these two cases are considered as phenocopies

There is strong support for the assumption that the AS is regulated by a single autosomal locus acting in a recessive manner. First of all, the pedigree structure of the affected calves in Simmental can best be explained by a recessive mode of inheritance. The paternal branch of the pedigree could be traced back to one sire, SEMPER, for all affected calves, the maternal branch in the majority of the cases. Inbreeding loops over a few generations are present in several pedigrees of affected calves, e.g. cases P3364 and P1787 (Figure 2). Sex-dependent inheritance can obviously be excluded and a dominant mode with reduced penetrance seems to be unlikely. Secondly, the experimental matings resulted in 4 affected and 25 unaffected fetuses, a result that most closely resembles the expectation of a recessive mode of inheritance. Thirdly, the occurrence of cases corresponded well with the numbers expected under the assumption of a recessively acting mutation. We tested this on the progeny of ROMEL, the largest dataset available from one carrier. We analyzed the period from the beginning of the recording system for malformations to May 2007. In that period 44,170 calves were born that were sired by ROMEL. From these, 662 were considered as risk pairings, i.e. the mother had a risk of 0.5 to be a carrier (i.e. one of the grandparents was an obligate carrier) and 35 calves out of these were diagnosed as affected. Since it is expected that about 1/2 to 1/3 of the affected calves were recorded, this result is very close to the expected 1:7 ratio of affected to unaffected calves. Moreover, these findings are concordant with the historical description of the arachnomelia syndrome in Simmental [2] and the analyses of cases in Brown Swiss [5]. Finally, when applying linkage analyses using microsatellite markers, evaluations with a model assuming recessive autosomal inheritance gave the highest lod scores (Buitkamp et al., in preparation).

### Allelic frequency of carriers in the present Simmental cow population

Since the arachnomelia syndrome-allele was passed to the current population through two parental lines (REXON and EGEL) and the main carriers are known, it is possible to estimate the frequency of the disease allele by an allele-counting method [10]. The allelic frequency was calculated for all cows from the breeding population who were alive in June 2007. In 10.4 percent of the pedigrees of 540,725 cows an identified carrier was found and the probability that individual cows were carrier of the arachnomelia syndrome-allele was calculated. E.g. in 14,740 and 41,032 cases a known carrier appeared as sire and grandsire, respectively. In these cases the probability of transmitting the allele is 50 and 25 percent, respectively, if no further carrier is present in the two generation pedigree. The averaged rate of the arachnomelia syndrome carriers based on known carriers over all cows alive in Bavarian Simmental was 3.32 percent.

Using this approach, the frequency of carriers was calculated for each year (always based on the actual datasets from August 2008) from 2003 to 2008 (Figure 1). The calculations were done twice, considering all known carriers together, and also by using only ROMEL as a carrier to show the numeric contribution of his progeny (Figure 1). For these analyses, the sires that are designated to be non-carriers by the number of risk pairings without having a case or the indirect gene test are set as non-carrier. Therefore, these frequencies are slightly lower than the initial frequency estimate of 3.32 percent.

## Conclusion

The cases of malformed Simmental calves presented here showed the same morphology described in the arachnomelia syndrome in Brown Swiss [e.g. [3]], even though there is a certain morphological variation from mild to severe malformations. The main findings, brachygnathia inferior and convex frontal bone of the face, deformation of vertebrae, and dysplasia of the limbs, namely the diaphyses of metatarsus and -carpus and the fetlocks, can best be explained by irregularly developed bone structure at the corresponding locations.

Without pathological examination it is difficult to distinguish the arachnomelia syndrome from other malformations of the limbs. Therefore, low numbers of cases in Simmental probably passed unrecognized before 2005. In that year the allelic frequency of the disease in the cow population increased sharply because some sires that had been carriers of the mutation had become very popular 2–4 years before.

The identification of a common ancestor, the results from the experimental matings and the analyses of numbers of cases from risk matings strongly support the hypothesis of an autosomal recessively inherited disease. Furthermore, this assumption is concordant with the historical description of the syndrome in Simmental and Brown Swiss. The allelic frequency of the arachnomelia syndrome in the current population is well above 3 percent and a substantial number of progeny from known carriers with superior genetic merit shall be used as sires during the next years. Therefore, a control system has to be established and the arachnomelia syndrome-gene should be mapped as a prerequisite for the development of an indirect gene test for carrier identification. The availability of pathologically well characterized cases from the field and from the ET-generated full-sib families will be an excellent material for a genetic mapping procedure.

## Methods

### Recording system for congenital malformations

A system for monitoring inherited congenital malformations in Bavarian cattle populations was established by the Institute for Animal Breeding of the Bavarian State Research Centre for Agriculture (ITZ) in cooperation with the Bavarian milk recording organization (LKV) [11]. In short, a questionnaire was developed for detailed recording of malformed calves. The malformation was described according to its location (e.g. head, legs) and its characteristics (e.g. hernia). The standardized data were stored in a database at the LKV, that is evaluated monthly for a potential genetic background of malformations.

Sires that fit into the pedigree (progeny of REXON or EGEL) with at least one affected calf with confirmed paternity were defined as obligate carriers and marked in the breeding information system [7]. In cases without connection to the pedigree and only one recorded calf the number of "risk pairings" (matings to cows where at least one parent is a known carrier, enabling the calculation of the probability for the occurrence of cases) was calculated. When the probability that a case occurs was above 99% for the sire in question the case was considered to be a phenocopy. The number of calves affected by the arachnomelia syndrome and their parentage is routinely published [8].

### Pathological examinations

Pathological examinations followed standard procedures. Calves were photographed and size and weight measurements were recorded. Tissue specimens from the condyle (epiphysis) and from the diaphysis of the femur were collected for histological examination. Specimens were fixed in 10% formalin and kept in a decalcifying solution (Ossafixonafor) for 24 hours. Thereafter, specimens were processed in an automated embedding system, sectioned at 4–6 microns and finally stained with haematoxyline and eosin.

### Experimental matings and embryo transfer

Known carriers of the arachnomelia syndrome (seven cows that had produced at least one affected calf) were brought to the facilities of the ITZ for embryo transfer (Table 1). Late morulae and blastocysts collected on day 7 (day 0 = estrus) from superovulated donor cows were nonsurgically transferred to heifers [12].

### Mode of inheritance and allele frequency

The pedigree of all cases was constructed from the pedigree that is used for the joint breeding evaluation of Germany and Austria. The graphical presentation of the pedigree was performed with the Pedigraph TM software [13]. The allelic frequency of the AS mutation in the current cow population was estimated from ancestors with known genotypes following the allele-counting method [10]. For this reason two generation pedigrees of herd book cows in Bavarian Simmental were analyzed for obligate carriers. We considered all cows that were alive in June 2007 and included in the herd book. All animals were bred by the use of artificial insemination.

### Statistical analyses

The non parametric Mann-Whitney-Test was performed using SPSS Version 14.0, the Chi-square test was performed using R 2.4.0 [14].

## Authors' contributions

JB drafted the manuscript and analyzed the pedigrees. BL conceived the monitoring system for inherited diseases. RE extracted the data from the database and estimated the allelic frequencies of the arachnomelia syndrome. HR and MW performed the embryo collection, transfer and recorded the morpho-metrical data of the experimental matings. BS examined the calves pathologically. NM and KG participated in study design and coordination and critically revised the manuscript.

All authors read and approved the final manuscript.

## References

[B1] Buitkamp J, Götz K-U (2006). Die Suche läuft. Fleckvieh.

[B2] Rieck GW, Schade W (1975). Arachnomelia (spider limbs), a new hereditary fatal malformation syndrome of cattle. Dtsch Tierarztl Wochenschr.

[B3] Testoni S, Gentile A (2004). Arachnomelia in four Italian brown calves. Vet Rec.

[B4] Brem G, Wanke R, Hondele J, Dahme E (1984). Occurrence of the arachnomelia syndrome in Bavarian Brown-Swiss × Braunvieh breed population in Bavaria. Berl Münch Tierärztl Wochenschr.

[B5] König H, Galliard C, Chavaz J, Hunziker F, Tontis A (1987). Prüfung von Schweizer Braunvieh-Bullen auf das vererbte Syndrom der Arachnomelie und Arthrogrypose (SAA) durch Untersuchung der Nachkommen im Fetalstadium. Tierärztl Umsch.

[B6] Schneeberger M, Stricker C (1985). Züchterische Aspekte der Spinnengliedrigkeit. KB-Mitteilungen, Schweiz.

[B7] Bayerische Zuchtwert-Informationen, BaZI-Rind. http://www.lfl.bayern.de/bazi-rind/.

[B8] Spinnengliedrigkeit (Arachnomelie) beim Fleckvieh. http://www.lfl.bayern.de/internet/stmlf/lfl/itz/rind/15200/index.php.

[B9] Geldermann H, Pieper U, Weber WE (1986). Effect of misidentification on the estimation of breeding value and heritability in cattle. J Anim Sci.

[B10] Allaire FR, Lucas JL, Secrist NL (1982). Estimation of frequency for recessive genes based on known ancestral genotypes. J Dairy Sci.

[B11] Untersuchung zu Missbildungshäufigkeit beim Fleckvieh. Aufbau eines Datenerfassungs- und Warnsystems beim Fleckvieh. http://www.lfl.bayern.de/itz/rind/32341/linkurl_0_3_0_0.pdf.

[B12] Reichenbach H-D (2003). Embryo transfer and cryopreservation in cattle: practical considerations. Acta Scientiae Veterinariae.

[B13] Pedigraph. http://animalgene.umn.edu/pedigraph/.

[B14] The R Project for statistical computing. http://www.r-project.org.

